# Genistein and Sex Hormone Supplementation Modulated Hepatic PPARα, δ, and γ Subtypes and STAT1 Expressions in a NASH Rat Model with Bilateral Orchidectomy

**DOI:** 10.3390/biomedicines12030483

**Published:** 2024-02-21

**Authors:** Fatist Okrit, Maneerat Chayanupatkul, Prasong Siriviriyakul, Natcha Wanpiyarat, Duangporn Werawatganon

**Affiliations:** 1 Center of Excellence in Alternative and Complementary Medicine for Gastrointestinal and Liver Diseases, Department of Physiology, Faculty of Medicine, Chulalongkorn University, Bangkok 10330, Thailand; fatist.okrit@gmail.com (F.O.); maneeratc@gmail.com (M.C.); fmedpsr@gmail.com (P.S.); 2 Department of Pathology, Faculty of Medicine, Chulalongkorn University, Bangkok 10330, Thailand; natchawanpi@gmail.com

**Keywords:** genistein, sex hormone, peroxisome proliferator-activated receptors (PPARs), orchidectomy (ORX), high-fat high-fructose diet (HFHF), non-alcoholic steatohepatitis (NASH)

## Abstract

Nonalcoholic steatohepatitis (NASH) is a progressive form of nonalcoholic fatty liver disease (NAFLD) that is characterized by hepatic inflammation and steatosis. Currently, limited data exist regarding the risk of NASH in transgender women and the treatment options for this particular population. The use of testosterone supplementation is unfavorable for transgender women, and estrogen supplementation is linked to an increased risk of breast cancer; thus, an isoflavone derivative compound known as “genistein” could serve as a viable substitute for a hormone supplement in this context. The purpose of this study was to investigate the treatment effects and mechanisms of actions of genistein and sex hormones in orchidectomized (ORX) rats with nonalcoholic steatohepatitis induced via a high-fat high-fructose diet (HFHF) model. Male *Sprague-Dawley* rats (n = 42) were randomly assigned into seven groups; control, ORX + standard diet, HFHF, ORX + HFHF, ORX + HFHF diet + testosterone (50 mg/kg body weight (BW) once weekly), ORX + HFHF diet + estradiol (1.6 mg/kg BW daily), and ORX + HFHF diet + genistein (16 mg/kg BW daily). The duration of the study was 6 weeks. Some parts of liver tissue were used for histological examination by H&E staining. The determination of fat accumulation was performed using Oil Red O staining. *SREBP1c* and *FAS* gene expression were quantified using real-time PCR technique. The levels of all types of peroxisome proliferator-activated receptors (PPARs; α, δ, γ), proteins, and signal transducer and activator of transcription 1 (STAT1) signaling pathway were determined by both immunoblotting and immunohistochemistry. Rats in the ORX + HFHF group had the highest degree of hepatic steatosis, lobular inflammation, and hepatocyte ballooning, and showed higher levels of genes related to de novo lipogenesis, including *SREBP1c* and *FAS*. The expression of PPARγ and STAT1 were upregulated, while the expression of PPARα and PPARδ were downregulated in the ORX + HFHF group. Testosterone, estradiol and genistein treatments improved NASH histopathology together with the reversal of all types of PPAR protein expressions. Interestingly, genistein decreased the levels of STAT1 protein expression more than those of testosterone and estradiol treatment. Genistein and sex hormone treatment could ameliorate NASH through the upregulation of PPARα, and PPARδ, and the suppression of PPARγ and STAT1 expression.

## 1. Introduction

Non-alcoholic steatohepatitis (NASH) is a morphological pattern of hepatic injury and a more serious form of non-alcoholic fatty liver disease (NAFLD). The percentage of NAFLD patients who had NASH were reported to be 20–30% and 10–20% in Western and Asian countries, respectively [1]. Nowadays, the occurrence of NASH is becoming a big global problem as it is associated with manifestations of metabolic syndromes, such as obesity and insulin resistance, and an increase in carbohydrate and fat consumption [2,3]. It is well accepted that consuming excessive amounts of high-calorie food, particularly saturated fatty acids and simple sugars, increases the risk of obesity and metabolic syndromes [4]. Therefore, a diet rich in fat and fructose (HFHF) has been commonly employed to simulate NASH in vivo [5].

Testosterone deficiency has been linked to the increased prevalence of diabetes, obesity, NAFLD, and NASH [6]. A previous transcriptomic analysis demonstrated that testosterone deficiency heightened the severity of high-fat diet (HFD)-induced NAFLD through the impairment of lipid metabolism, and the increment in inflammatory activity, oxidative stress, and apoptosis [7]. Taken together, the prevalence of male-to-female (MTF) transgender individuals has been on the rise in Thailand over the last two decades [8]. An orchidectomy is a medical procedure to remove both testicles that is utilized to eliminate the effect of endogenous testosterone [9]. Additionally, a clinical study demonstrated that orchidectomy played a protective role in metabolic health in transwomen [10]. In contrast, an animal study showed the adverse effect of orchidectomy on hepatic steatosis development [7]. However, the impact of orchidectomy on NASH is still a topic of discussion, lacking supported data on the risk of NASH in transgender women and effective treatment for this particular population.

Male and female sex hormones have been previously shown to attenuate varied NAFLD parameters [11], but very few studies have evaluated the effects of sex hormones on the NASH model induced using a high-fat high-fructose diet with bilateral ORX. This model will be developed to imitate transwomen with NASH condition. Male sex hormone supplements are not desirable in these patients and female sex hormones have been reported to increase breast cancer in transgender women [12]. Therefore, the natural soy product “genistein” is an attractive alternative to hormone replacement therapy. Genistein is an estrogen-like molecule that possesses antioxidant, anti-inflammation, and anti-fibrotic effects. Recent studies demonstrated that genistein and hormonal treatment may influence the progression of NASH through the actions of peroxisome proliferator-activated receptors (PPARs) [11,13,14,15]. However, the effects of genistein and sex hormone treatment on different types of PPAR and its roles in regulating hepatic lipid metabolism and inflammation remain uncertain. Furthermore, signal transducer and activator of transcription 1 (STAT1) signaling pathway, the intracellular signal transduction that plays a crucial role in inflammatory responses, can be activated by varied pathogenic factors, thus promoting the NASH progression [16,17]. In a mouse model with obesity, researchers found that obesity triggered the development of NASH through the activation of STAT1 signal transduction, while the inhibition of this STAT1 could improve NASH [18]. Thus, we hypothesized that STAT1 signaling pathway may also be a key regulator of inflammation in non-alcoholic steatohepatitis (NASH) induced via testosterone deficiency and HFHF diet.

Therefore, we imitated the occurrence of NASH in transgender women in a rat model by providing a high-fat high-fructose (HFHF) diet with orchidectomy (ORX) and evaluating whether genistein, a phytoestrogen, and standard sex hormone supplement could improve NASH through the modification of all types of peroxisome proliferator-activated receptors (PPARs; α, δ, γ) and signal transducer and activator of transcription 1 (STAT1) signaling pathway.

## 2. Materials and Methods

### 2.1. Animals and Experimental Procedures

Seven-week-old male Sprague-Dawley rats (42 in total, body weight 210–250 g) were obtained from National Laboratory Animal Center, Mahidol University, Thailand, and used in this study. All rats were housed in cages under a standard condition (room temperature at 25 °C with 12:12 light–dark cycle) and fed with a standard diet for one week for the purpose of acclimatization. After the acclimatization period, rats were randomly allocated into three major groups: (i) rats fed with a standard diet (control group, n = 6); (ii) rats fed with high-fat high-fructose diet (HFHF group, n = 6); and rats that underwent bilateral orchidectomy (ORX) (ORX group, n = 30). Then, rats in the ORX group were sub-divided into five subgroups (six rats in each group): (iii) ORX rats fed with a standard diet (ORX group), (iv) ORX rats fed with an HFHF diet (ORX + HFHF group), (v) ORX rats fed with an HFHF diet and injected subcutaneously with testosterone at 50 mg/kg body weight once weekly (ORX + HFHF diet + Test group), (vi) ORX rats fed with an HFHF diet that have received estradiol by oral gavage at 1.6 mg/kg body weight once daily (ORX + HFHF diet + E2 group) and (vii) ORX rats fed with an HFHF diet that have received genistein at 16 mg/kg body weight daily by oral gavage (ORX + HFHF diet + Gen group). Testosterone enanthate dosage at 50 mg/kg BW (Bayer Pharma AG, BER, Germany) was derived from our pilot study. Estradiol valerate dosage at 1.6 mg/kg BW was derived from Li T and colleagues [19], having been proven to be effective in reducing obesity and vulvovaginal atrophy in ovariectomized rats. Additionally, genistein dosage at 16 mg/kg BW was based on our previous publications, which demonstrated its efficacy in improving NASH induced via OVX + HFHF diet in female rats [14,20].

Bilateral orchidectomy was performed to induce the state of testosterone deficiency in orchidectomized groups. In brief, ORX rats underwent a bilateral orchidectomy procedure using a scrotal approach while under anesthesia. Rats were sedated with 100% concentration of Isoflurane (Terrell, TX, USA) at dose 1.5–3% via precision vaporizer. An incision was made on the ventral side of the scrotum. The testicular fat pad was located and gently extracted through the incision line using blunt forceps. Subsequently, the contents of the testicles were exposed, and the primary blood vessels situated above the testicles were carefully tied off using sterile absorbable silk to prevent any bleeding. After the ligation, the testis and epididymis were cautiously excised, and the residual content of the testicular sac was returned into the scrotum. Finally, the skin was sutured with non-absorbable silk and aseptic was prepared over the area of surgery. The same procedures were duplicated on the contralateral side (Supplementary Figure S1).

A standard diet is composed of 6% fat, 47% carbohydrate, and 25% protein, which was provided for the control group. This diet was purchased from Perfect Companion Group Co., Ltd., Thailand. An HFHF diet was formulated based on a prior research [14]. The composition of the HFHF diet comprised 55% fat from vegetable oil, 20% carbohydrate from fructose and 15% starch, and 10% protein from egg albumin. After the adaptation period, all other ORX + HFHF groups were given HFHF diet to induce NASH pathology.

The duration of the experiment was 6 weeks. In the control, ORX, and ORX + HFHF groups, rats were administered 1 mL of 0.1% dimethyl sulfoxide once daily as a vehicle control. All rats were provided unrestricted access to food and water ad libitum for six weeks. Lastly, rats were weighed weekly. At the end of the experiment, the rats were subjected to an overnight fast (6–8 h) and then euthanized using an intraperitoneal injection of thiopental sodium at a dosage exceeding 50 mg/kg body weight. Following euthanasia, the entire liver tissue was promptly collected and rinsed with ice-cold normal saline solution (NSS). In each rat, the seminal vesicle was gathered, weighed, and examined to verify the successful bilateral orchidectomy. Liver tissue was collected for histological study using hematoxylin and eosin (H&E) staining. The determination of hepatic lipid deposition was examined using Oil Red O staining, and mRNA levels of FAS and SREBP1-c were measured using real-time qPCR analysis. The levels of PPARs—PPARα, PPARδ, PPARγ, p-STAT1, and t-STAT1 proteins—were determined using immunoblotting. Immunohistochemistry was also performed on the paraffin-embedded tissue to assess all PPAR subtypes (α, δ, and γ) expression.

### 2.2. Histopathological Analysis

In preparation for histological analysis, liver tissues were preserved in 10% formalin overnight, then transferred to paraffin for embedding. The tissues were subsequently cut into sections with a thickness of 5 µm before being stained using the hematoxylin and eosin (H&E) method. All liver sections were assessed and graded under a light microscope by an experienced pathologist who was blinded to all experimental samples. Three key features of NAFLD activity score (NAS)—hepatic steatosis, lobular inflammation, and hepatocyte ballooning—were scored based on the Brunt criteria [21].

Following Brunt’s criteria, steatosis (grade 0–3) was categorized as 0 for < 5%, 1 for < 33%, 2 for 33–66%, and 3 for > 66% of fat deposition in hepatocytes. Inflammation (grade 0–3) was rated as 0 for normal, 1 for mild, 2 for moderate, and 3 for severe. Hepatocyte ballooning was assessed as 0 for no ballooning, 1 for a few balloon cells, and 2 for many cells or prominent ballooning.

### 2.3. Hepatic Lipid Deposition by Oil Red O Staining

All procedures including embedding, cutting, and sectioning were performed under cryostat microtome (Dakewe, Shenzhen, China) at −20 °C. Briefly, a small piece of frozen liver sample was thawed and embedded via OCT solution on a tissue cryomold. A 5-µm-thickness of tissue section was sectioned and pasted on adhesive microscope slide (Matsunami, Japan). Subsequently, each liver section was immediately stained with fresh Oil Red O (Sigma, St. Louis, MO, USA) working solution. After that, sections were counterstained with hematoxylin, rinsed with running tap water, air dried, mounted, and covered with cover slip glasses to avoid bubble infiltration. Ten different fields of each sample at 40× resolution were randomly captured under a light microscope (Nikon Eclipse E200, Melville, NY, USA) installed with Nikon DS-Fi3 camera.

### 2.4. Total RNA Preparation, cDNA Synthesis and Quantitative RT-PCR Technique

Total RNA was isolated from each rat’s liver with PurelinkTM RNA mini kit plus Trizol reagent (Invitrogen, Carlsbad, CA, USA) and treated with DNase I treatment (Thermo Scientific, Bannockburn, IL, USA). Then, the iScript Reverse Transcription Supermix (BioRad, Hercules, CA, USA) was used for the first-strand complementary DNA (cDNA) synthesis using 1 µg of total RNA. The volume of reaction was set at 25 °C for 5 min (priming), and then 46 °C for 20 min (reverse transcription), followed by RT inactivation process at 95 °C for 1 min. The profiles of gene expression level were evaluated using SsoAdvanced Universal SYBR Green Supermix (BioRad, Hercules, CA, USA); qPCR MasterMix was used for the running process per manufacturer’s protocol. The QuantStudio 6 Flex Real-Time PCR machine (Applied Biosystems, Foster City, CA, USA) detection was used for the quantitative RT-PCR analysis. The thermal cycling conditions were set as followed: 95 °C for 5 min (1 cycle), 95 °C for 15 s, and 60 °C for 1 min (40 cycles). Beta actin (β-actin) was used as a housekeeping gene for mRNA quantification. The primer sequences used to measure the gene expression of interest are listed in Supplementary Table S1 [22]. Relative quantification of polymerase chain reaction analysis was used to calculate the quantity of target mRNA in term of CT values (using 2^−∆∆CT^ method) by comparing to the internal control.

### 2.5. Hepatic Immunohistochemistry Processes and Analysis

Tissue microarray (TMA) is a histopathological technique that allows simultaneous analyses of multiple tissue samples. It was initially introduced by Battifora H. in 1986 and has since been established as a valuable and efficient method for immunohistochemical (IHC) studies [23]. The recipient block was precisely pierced by Quick-Ray Automated Tissue Microarrayer (Diagnostic Technology Pty Ltd., Belrose, NSW, Australia) in a specific array pattern. Then, a hollow needle was applied to remove tissue cores of approximately 0.6 cm in diameter from the donor blocks and inserted them into the empty holes of recipient paraffin blocks. The area of which the tissue core is selected was marked on the corresponding H&E slide by an experienced pathologist. Finally, the TMA block was cut into thin slices and stained accordingly.

Immunohistochemistry was conducted to assess hepatic PPARα, PPARδ, and PPARγ expression across all experimental groups. Liver sections were briefly deparaffinized and rehydrated using varying concentrations of xylene and ethanol. Subsequently, antigen retrieval was carried out in a microwave using citrate buffer at pH 6.0. To avoid misinterpretation due to false positive staining, endogenous peroxidase activity was blocked using 3% hydrogen peroxide (H_2_O_2_). The slides were then exposed to the primary antibody PPARs (α, δ, and γ; Abcam, Cambridge, MA, USA) at 1:300 concentration for 1 h at room temperature. This was followed by incubation with a secondary antibody for 30 min. Positive staining appeared as a brown color after incubation with diaminobenzidine, and the slides were counterstained with hematoxylin. Histological slides from TMA were scanned using a whole slide scanner, specifically the Aperio ScanScope (Leica Biosystems Imaging, Inc., Deer Park, MD, USA). Digital images were captured and analyzed at a resolution of 40× with Aperio ImageScope software program version 12.4.6. Each TMA core was observed with a field size equivalent to a square area of 200 μm × 400 μm, totaling 0.020 mm^2^. Brown color in ten random fields from each sample was computed and presented as a ratio between the total number of positive pixels and the total number of pixels using Positive pixel count V9 algorithm.

### 2.6. Hepatic Peroxisome Proliferator-Activated Receptor Alpha, Delta, and Gamma Subtypes (PPAR α, δ, and γ) and Phospho-STAT1 Protein Expression by Immunoblot Analysis

Small pieces of frozen liver tissues were homogenized and centrifuged. The supernatant was collected to calculate the total protein concentrations using Pierce BCA assay kit (Thermo Scientific, Bannockburn, IL, USA). Then, 30 µg of tissue lysate was equally loaded on 10% sodium dodecyl sulfate polyacrylamide gel electrophoresis (SDS-PAGE) in which protein migrated in an electric field according to their molecular weight (80 v for stacking gel: 30 min, 120 V for separating gel: 90 min). Proteins were then transferred onto 0.45 µm polyvinylidene fluoride (PVDF) (Merck, Millipore, Billerica, MA, USA) membrane via a semi-dry method. Blots were placed in a solution containing 1% bovine serum albumin (BSA) with phosphate buffer saline plus 0.1% Tween 20 (PBST) for 1 h at a room temperature to block non-specific proteins. Subsequently, the membrane was incubated with primary antibodies; PPARα (1:1000), PPARγ (1:1000), PPARδ (1:1000), p-STAT1 (1:2000), and t-STAT1 (1:1000) (Abcam and cell signaling, USA) at 4 °C overnight. After that, the membrane was washed for 10 min with PBST three times and then incubated with horseradish peroxidase (HRP)-conjugated secondary antibodies (1:10,000, goat anti-rabbit IgG) at room temperature for 1 h according to the manufacturer’s instructions. At the final step, membranes were washed three times for 10 min each with PBST again before visualization. Cyclophilin B (CPB) (1:10,000, Abcam, Waltham, MA, USA) was used as a reference protein. Levels of PPARα, PPARδ, PPARγ and p-STAT1 protein expression were reported as the relative protein expression between band densities of each protein to CPB. For the visualization, signals of all target proteins were generated using enhanced chemiluminescence kit (ECL; BioRad, NSW, Australia) on ChemiDoc^TM^ Touch Imaging System (BioRad laboratories, Hercules, CA, USA). The band density values were quantified using Image Lab Software version 6.1.

### 2.7. Statistical Analysis

SPSS software version 22.0 for Windows (SPSS, Inc., Chicago, IL, USA) was used for data analysis. The data were presented as the mean ± standard deviation (SD). Continuous variables were compared between groups using one-way analysis of variance (One-way ANOVA) and Tukey’s HSD post-hoc test. Descriptive statistics were used for histological examination. Statistical significance was indicated by a *p*-value less than 0.05 (*p* < 0.05).

## 3. Results

### 3.1. Rats’ General Appearance and Delta Body Weight Changes

**Figure 1:**
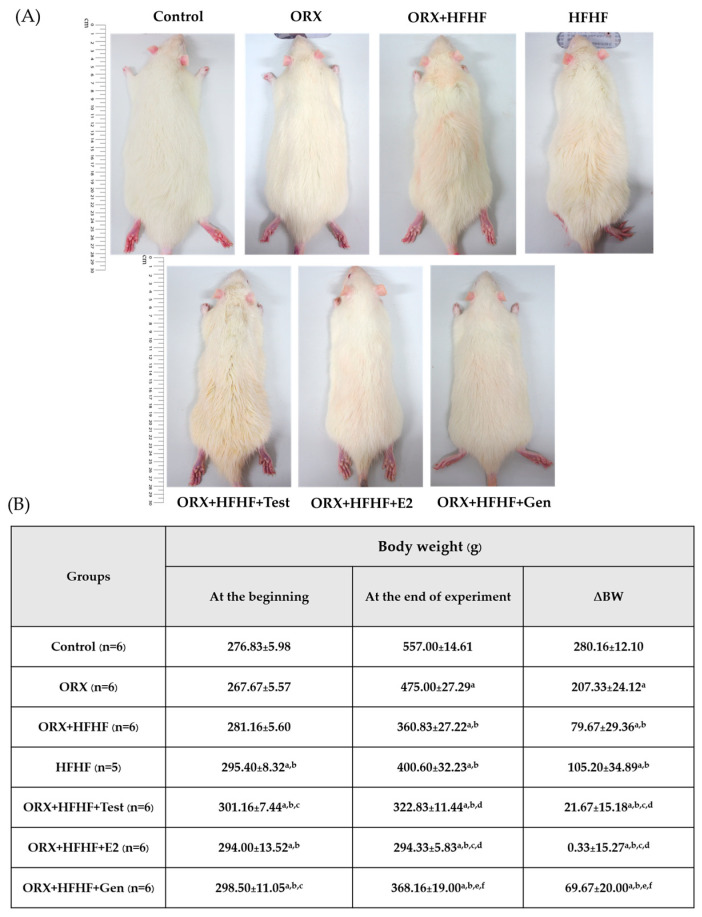
(**A**) Images illustrated the general appearance of the rats in all experimental groups. (**B**) Average body weight changes (delta) in all experimental groups (n = 6 per group, except the HFHF group, n = 5). Data are expressed as mean ± SD. ^a^: *p* < 0.05 when compared with the control group, ^b^: *p* < 0.05 when compared with the ORX group, ^c^: *p* < 0.05 when compared with the ORX + HFHF group, ^d^: *p* < 0.05 when compared with the HFHF group, ^e^: *p* < 0.05 when compared with the ORX + HFHF + Test group and ^f^: *p* < 0.05 when compared with the ORX + HFHF + E2 group.

Rats’ body weights were recorded once weekly throughout the experiment (Supplementary Figure S2). The delta body weight of the rats between the start and the end of the experiment was calculated to observe the body weight changes and monitor treatment effects (delta body weight = BW at the end-BW at the start). At the end of the study, the delta body weights of the rats in HFHF and ORX + HFHF groups were lower when compared to those control and ORX fed standard diet groups (105.2 ± 34.9 vs. 79.67 ± 27.39 vs. 280.16 ± 12.10 vs. 207.33 ± 24.12, respectively; *p* < 0.05). Moreover, the delta body weights after the treatments were even lower in testosterone- and estradiol-treated groups as compared to control, ORX, ORX + HFHF, and HFHF groups (21.67 ± 15.18 vs. 0.33 ± 15.27 vs. 280.16 ± 12.10 vs. 207.33 ± 24.12 vs. 105.2 ± 34.9 vs. 79.67 ± 27.39, respectively; *p* < 0.05). In addition, the delta body weights in genistein treated rats were significantly lower when compared to control and ORX groups (69.67 ± 20.00 vs. 280.16 ± 12.10 vs. 207.33 ± 24.12, respectively, *p* < 0.05), but were similar to those in testosterone and estradiol treatment (69.67 ± 20.00 vs. 21.67 ± 15.18 vs. 0.33 ± 15.27, respectively; *p* ≥ 0.05) (Figure 1).

### 3.2. The Confirmation of Complete Bilateral Orchidectomy

**Figure 2:**
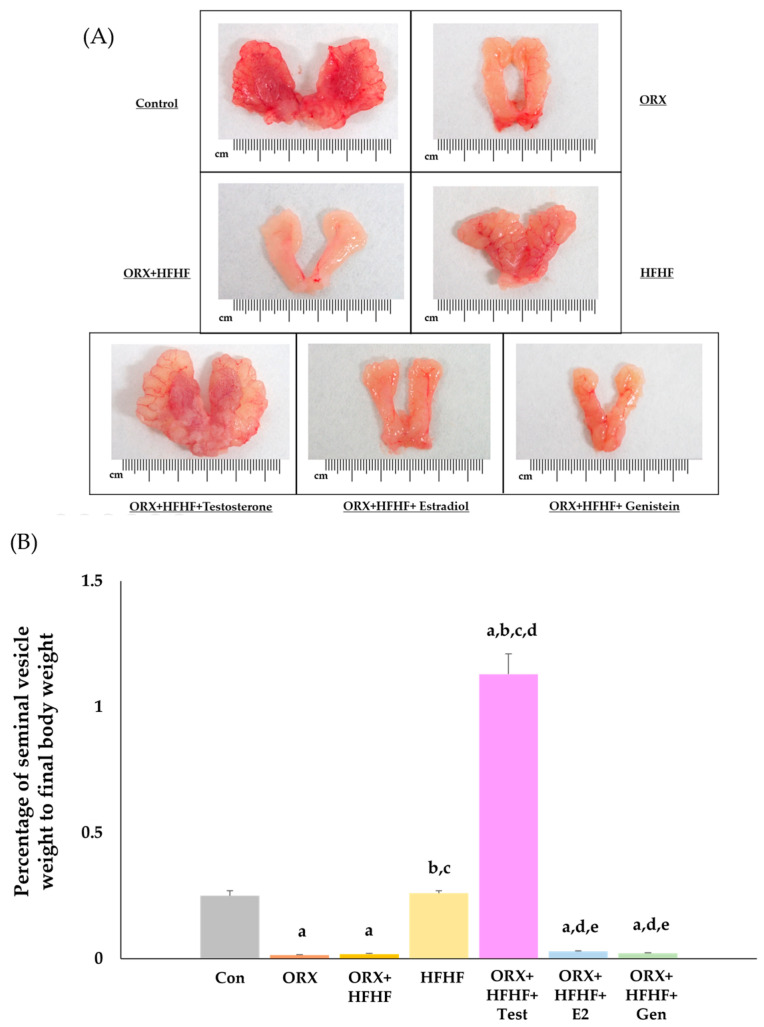
(**A**) Photographs of seminal vesicles in each experimental group. The atrophy of seminal vesicle was markedly seen in testosterone-deficient rats after orchidectomy, while the presence of testosterone maintained normal size and growth. (**B**) Rat percentage of seminal vesicle weight to final body weight in all experimental groups (n = 6 per group, except the HFHF group, n = 5). Data are presented in mean ± SD. ^a^: *p* < 0.05 when compared with the control group, ^b^: *p* < 0.05 when compared with the ORX group, ^c^: *p* < 0.05 when compared with the ORX + HFHF group, ^d^: *p* < 0.05 when compared with the HFHF group, and ^e^: *p* < 0.05 when compared with the ORX + HFHF + Test group.

The general appearance and the percentage of seminal vesicles to final body weight were measured to confirm the completion of bilateral orchidectomy (ORX) because testosterone has an influence on seminal vesicle growth and development. Normal features of seminal vesicles were seen in control and HFHF groups, in which testosterone was still present. Due to the use of high-dose testosterone injection (50 mg/kg of testosterone enanthate), the size of seminal vesicles was higher in the ORX + HFHF + Testosterone group when compared to control and HFHF groups. Contrarily, the absence of testosterone in other ORX groups led to smaller size of seminal vesicular glands as compared to groups in which testosterone was still present (Figure 2A).

In this study, we observed that percentage of seminal vesicle weight to final body weight was significantly lower in ORX and ORX + HFHF groups than in the control group (0.014 ± 0.002 vs. 0.018 ± 0.003 vs. 0.25 ± 0.02, respectively; *p* < 0.05). Likewise, significant reductions in that percentage of seminal vesicle weight to final body weight were seen in ORX + HFHF + Estradiol and ORX + HFHF + Genistein groups when compared to the control group (0.029 ± 0.002 vs. 0.022 ± 0.001 vs. 0.25 ± 0.02, respectively; *p* < 0.05). Administration of testosterone enanthate could completely restore the percentage of seminal vesicle weight to final body weight in ORX + HFHF + Testosterone group when compared to that in ORX + HFHF group (1.13 ± 0.08 vs. 0.018 ± 0.003, respectively; *p* < 0.05). On the contrary, both estradiol and genistein supplement did not affect seminal vesicle. Thus, these data confirmed that the bilateral orchidectomy caused testosterone depletion (Figure 2B).

### 3.3. Liver General Appearance and Histopathology

**Figure 3:**
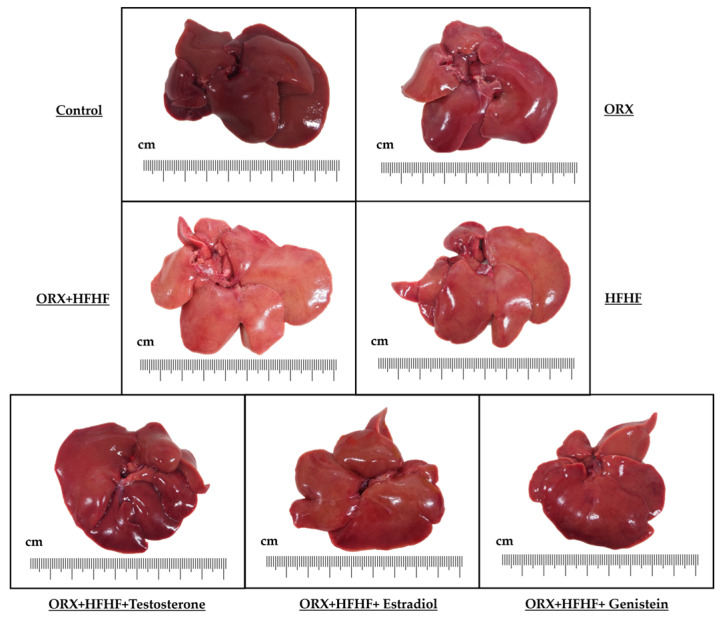
Images of gross liver morphology in each experimental group. The livers’ hue appeared slightly yellowish and had a pale-yellowish quality in both HFHF and ORX + HFHF groups when exposed to a high-fat high-fructose (HFHF) diet. Livers in all treatment groups displayed dark brown in color. ORX: orchidectomy; HFHF: high-fat high-fructose diet.

Morphological examination of liver was captured for each animal using an Olympus OMD-E-M5 camera. Gross liver examination showed that livers in the control and ORX groups were fresh-reddish in color and appeared normal. In contrast, livers in HFHF and ORX + HFHF groups were enlarged with soft and pale yellowish color which likely indicated hepatic steatosis (more obvious in the ORX + HFHF group). Genistein and sex hormone supplementation considerably improved liver morphology as compared with ORX + HFHF and HFHF groups (Figure 3).

**Figure 4:**
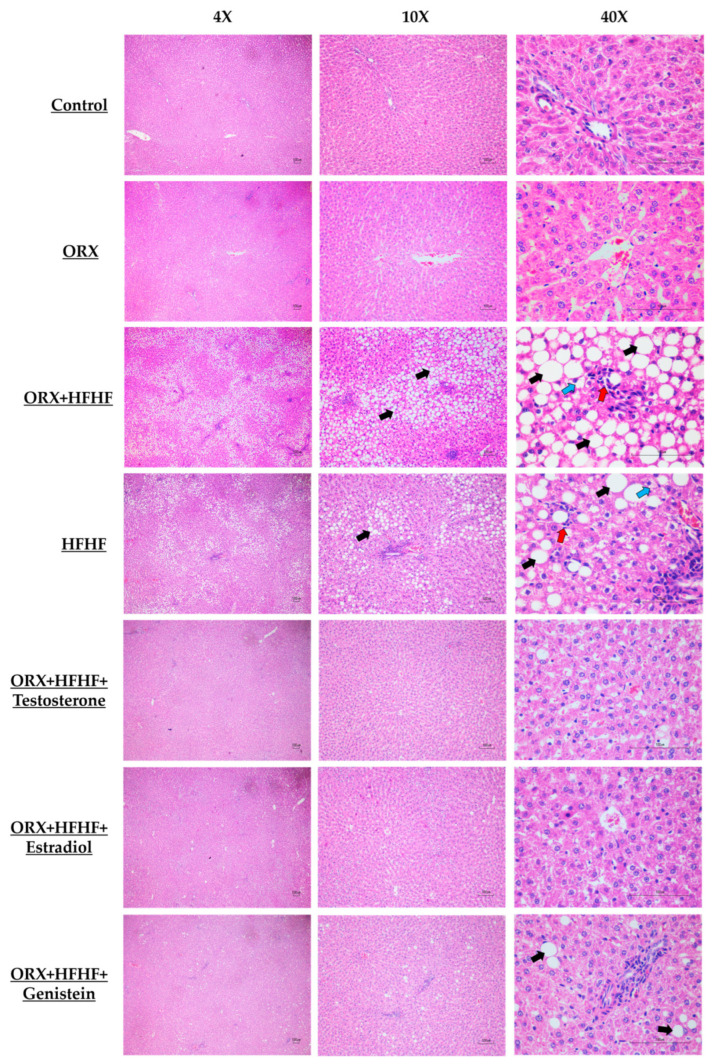
Representative photomicrographs of liver histopathology in all experimental groups using hematoxylin-eosin (H&E) staining (4×, 10× and 40× magnifications; scale bar, 100 µm; n = 6 per group, except the HFHF group, n = 5). Black arrows indicate macrovesicular steatosis; blue arrows indicate hepatocellular ballooning; red arrows indicate inflammation. Histological features of hepatic steatosis and hepatocellular ballooning were apparently seen in the ORX + HFHF group. Testosterone, estradiol, and genistein treatments could alleviate it when compared with the ORX + HFHF group.

Hematoxylin and eosin (H&E) sections in each experimental group were demonstrated in Figure 4. In the control group, liver histology appeared normal. In the ORX + standard diet group, some degree of lobular inflammation and hepatocyte ballooning was seen as compared with the control group. This finding indicated the protective role of testosterone on hepatocyte injury. In the HFHF group, a higher degree of steatosis, inflammation and hepatocyte ballooning was observed when compared with the control and ORX + standard diet groups. All NASH histological features were most severe in the ORX + HFHF diet group when compared to control, ORX + standard diet, and HFHF groups. Testosterone, estradiol, and genistein administration could attenuate all NASH histological features comparing with the ORX + HFHF group.

A summary of histopathological parameters in each group is provided in Table 1. The summation of NASH activity score was significantly higher in the HFHF and ORX + HFHF groups as compared with the ORX and control groups (4.20 ± 0.20 vs. 5.50 ± 0.42 vs. 2.00 ± 0.44 vs. 0.83 ± 0.30, respectively; *p* < 0.05). Administration of testosterone, estradiol, and genistein reduced NASH activity score when compared with the ORX + HFHF and HFHF groups (1.83 ± 0.30 vs. 2.00 ± 0.00 vs. 3.50 ± 0.34 vs. 5.50 ± 0.42 vs. 4.20 ± 0.20, respectively; *p* < 0.05).

### 3.4. Hepatic Lipid Deposition and Lipogenic Genes Expression

**Figure 5:**
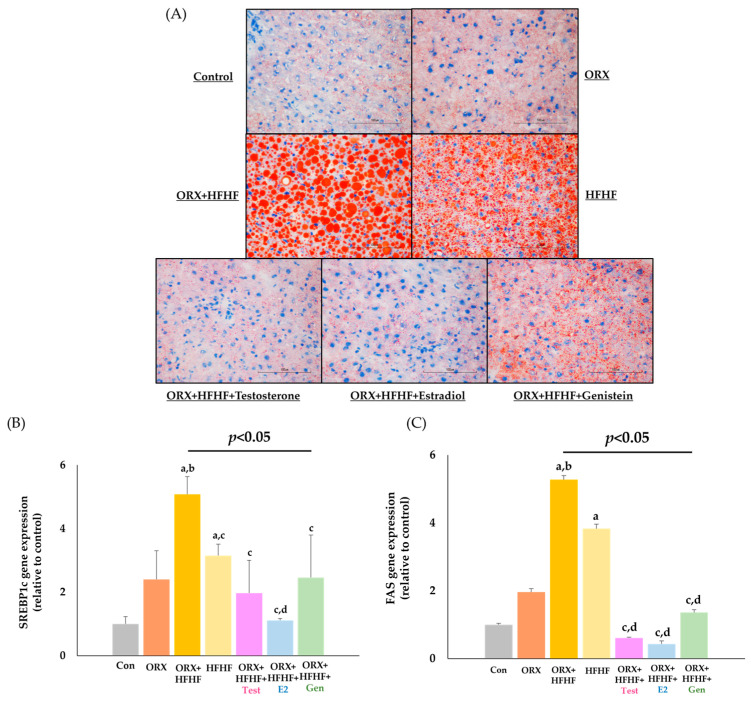
(**A**) The degree of hepatic lipid deposition in each experimental group via Oil Red O staining. Intracellular lipid droplets were greater in the ORX + HFHF group but decreased following the treatments. Original magnification is 40×. (**B**,**C**) The levels of SREBP1c and FAS gene expression in all experimental groups (n = 6 per group, except the HFHF group, n = 5). Samples were normalized to β-actin. The relative gene expression is expressed as mean ± SD. ^a^: *p* < 0.05 when compared with the control group, ^b^: *p* < 0.05 when compared with the ORX group, ^c^: *p* < 0.05 when compared with the ORX + HFHF group, ^d^: *p* < 0.05 when compared with the HFHF group.

Lipid droplets in frozen liver tissue sections were stained in red droplets with specific Oil Red O solution. Then, photomicrographs were visualized and taken under a regular light microscope at 40× magnification as presented in Figure 5A. A significant hepatic fat deposition was observed in the HFHF and ORX + HFHF groups when compared with the control and ORX groups. However, in the HFHF group, fat droplets were mostly microvesicular, whereas in the ORX + HFHF group, they were macrovesicular. After treatment with testosterone, estradiol and genistein, the degree of fat deposition was significantly reduced from that of the ORX + HFHF group. The percentage of positive Oil Red O staining area was provided in Supplementary Table S2.

Hepatic lipogenic genes expression was shown in Figure 5B,C. *SREBP1c* gene expression in the liver tissue was significantly higher in both the ORX + HFHF and HFHF diet groups compared to the control group (5.08 ± 0.56 vs. 3.15 ± 0.36 vs. 1.00 ± 0.23, respectively; *p* < 0.05). Moreover, the ORX + HFHF group exhibited a significant increase of *SREBP1c* gene expression when compared to the ORX + standard diet and HFHF groups (5.08 ± 0.56 vs. 2.40 ± 0.90 vs. 3.15 ± 0.36, respectively; *p* < 0.05). Treatment with testosterone, estradiol and genistein decreased *SREBP1c* gene expression as compared to the ORX + HFHF group (1.97 ± 1.03 vs. 1.11 ± 0.06 vs. 2.46 ± 1.34 vs. 5.08 ± 0.56, respectively; *p* < 0.05).

*FAS* gene expression in the liver tissue showed a slight increase in the ORX group, albeit not statistically significant when compared to the control group (1.96 ± 0.18 vs. 1.00 ± 0.27, *p* = 0.85), whereas *FAS* gene expression in the HFHF diet group was significantly increased when compared with the control group (3.83 ± 1.70 vs. 1.00 ± 0.27, *p* < 0.05). Likewise, ORX + HFHF rats had significantly higher *FAS* gene expression levels in comparison with those in the control and ORX groups (5.27 ± 1.22 vs. 1.00 ± 0.27 vs. 1.96 ± 0.18, respectively; *p* < 0.05). Testosterone, estradiol, and genistein administration significantly reduced the levels of *FAS* gene expression as compared to those of the ORX + HFHF and HFHF groups (0.61 ± 0.08 vs. 0.43 ± 0.05 vs. 1.36 ± 0.48 vs. 5.27 ± 1.22 vs. 3.83 ± 1.70, respectively; *p* < 0.05).

### 3.5. Hepatic PPARα, δ, γ and STAT1 Expression by Immonoblotting

**Figure 6:**
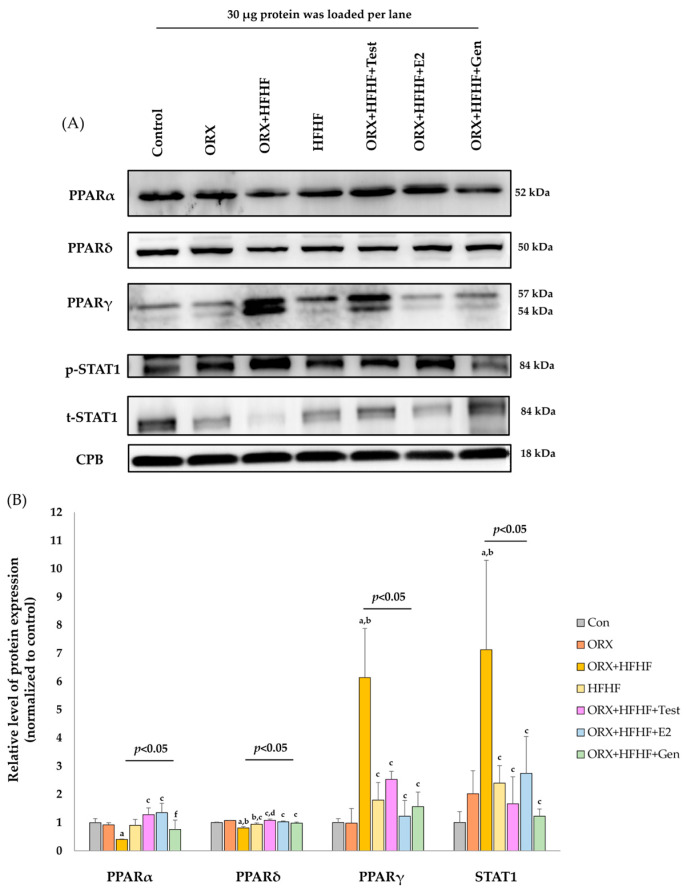
(**A**,**B**) Representative immunoblotting images for all PPAR subtypes (PPARs; α, δ, γ) and STAT1 protein expression. Bar graphs representing relative protein expressions of PPARα, PPAR δ, PPAR γ, *p*-STAT1 and t-STAT1 to CPB, respectively (n = 6 per group, except the HFHF group, n = 5). The ratio of each PPAR subtype and STAT1 was normalized to the control group and expressed as mean ± SD. ^a^: *p* < 0.05 when compared with the control group, ^b^: *p* < 0.05 when compared with the ORX group, ^c^: *p* < 0.05 when compared with the ORX + HFHF group, ^d^: *p* < 0.05 when compared with the HFHF group and ^f^: *p* < 0.05 when compared with the ORX + HFHF + E2 group.

Both immunoblot and immunohistochemical studies demonstrated similar patterns of protein expression for all PPAR subtypes (Figure 6). The protein levels of PPARα ratio to CPB were significantly downregulated in the ORX + HFHF group, while levels in both ORX and HFHF groups were slightly lower when compared to the control group (0.40 ± 0.02 vs. 0.92 ± 0.08 vs. 0.90 ± 0.21 vs. 1.00 ± 0.14, respectively; *p* < 0.05). A significant increase in PPARα expression was observed in testosterone- and estradiol- treated groups when compared with the ORX + HFHF group (1.28 ± 0.24 vs. 1.35 ± 0.33 vs. 0.40 ± 0.02, respectively; *p* < 0.05). PPARα expression had a non-significant trend toward increased expression in the genistein-treated group but remained significantly lower than that in the estradiol-treated group.

The protein levels of PPARδ ratio to CPB were slightly decreased in the HFHF group when compared with those of the ORX + standard diet and ORX + HFHF groups (0.94 ± 0.05 vs. 1.07 ± 0.01 vs. 0.81 ± 0.05, respectively; *p* < 0.05). In line with the HFHF group, PPARδ expression showed a significant decrease in the ORX + HFHF group as compared with the control and ORX + standard groups (0.81 ± 0.05 vs. 1.00 ± 0.02 vs. 1.07 ± 0.01, respectively; *p* < 0.05). After treatment with testosterone, estradiol and genistein, the reversal of PPARδ protein expression was observed comparing with the ORX + HFHF group (1.08 ± 0.05 vs. 1.03 ± 0.03 vs. 0.98 ± 0.04 vs. 0.81 ± 0.05, respectively; *p* < 0.05). Similarly, testosterone treatment significantly increased PPARδ levels when compared with the HFHF group (1.08 ± 0.05 vs. 0.94 ± 0.05, *p* < 0.05).

The protein levels of PPARγ ratio to CPB were significantly upregulated in the ORX + HFHF group as compared to those of control and ORX + standard diet groups (6.14 ± 1.74 vs. 1.00 ± 0.14 vs. 0.98 ± 0.52, respectively; *p* < 0.05). Similarly, PPARγ protein expression had an upward trend in the HFHF group as compared with control and ORX + standard diet groups (1.80 ± 0.62 vs. 1.00 ± 0.14 vs. 0.98 ± 0.52, respectively; *p* > 0.05), but was significantly lower than the ORX + HFHF group (1.80 ± 0.62 vs. 6.14 ± 1.74, *p* < 0.05). Treatment with testosterone, estradiol, and genistein could decrease the level of PPARγ expression compared with the ORX + HFHF group (2.53 ± 0.29 vs. 1.23 ± 0.55 vs. 1.57 ± 0.51 vs. 6.14 ± 1.74, respectively; *p* < 0.05).

Band densities of phospho and total STAT1 protein were calculated for the relative protein expression via normalization with protein cyclophilin B (CPB). In the ORX + HFHF group, protein levels of STAT1 were significantly upregulated compared to those of the control and ORX + standard diet groups (7.13 ± 3.17 vs. 1.00 ± 0.39 vs. 2.02 ± 0.82, respectively; *p* < 0.05). In the HFHF group, STAT1 protein level showed a similar pattern of protein expression, but was significantly lower when compared with the ORX + HFHF group (2.40 ± 0.62 vs. 7.13 ± 3.17, *p* < 0.05). After treatment with testosterone, estradiol, and genistein, STAT1 protein expression in the liver tissue decreased significantly as compared to the ORX + HFHF group (1.67 ± 0.95 vs. 2.75 ± 1.30 vs. 1.23 ± 0.25 vs. 7.13 ± 3.17, respectively; *p* < 0.05). 

### 3.6. Hepatic PPARα, δ, and γ Expression by Immunohistochemistry Detection

**Figure 7:**
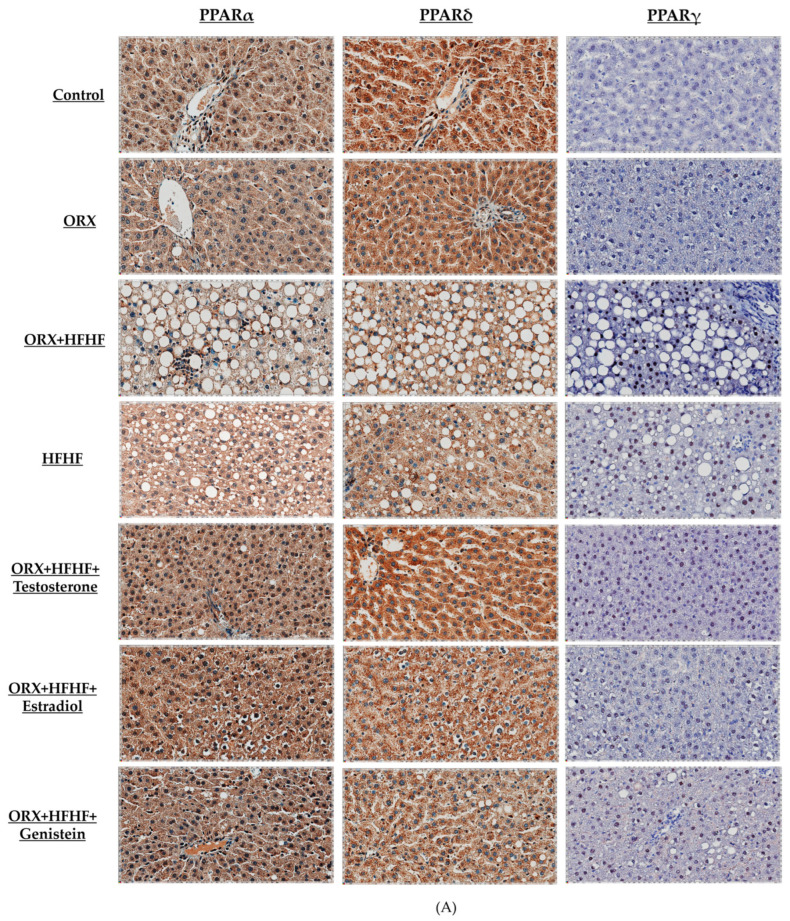

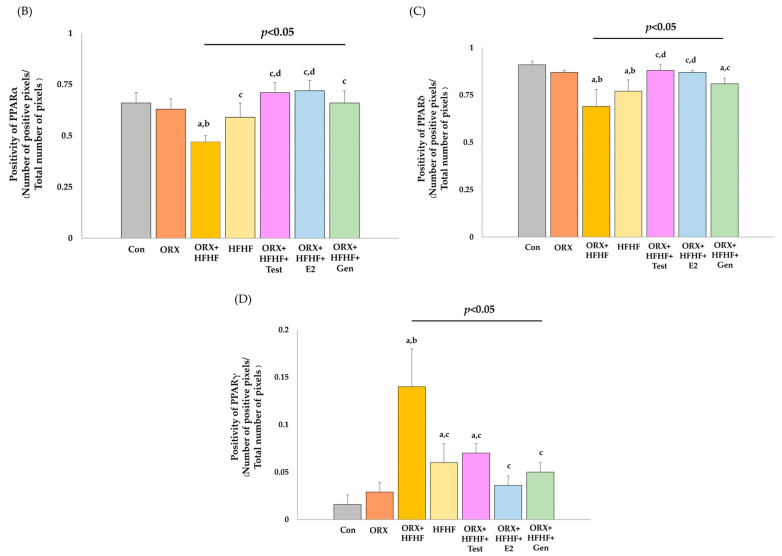
(**A**–**D**) Representative immunohistochemical images for all PPAR subtypes (PPARs; α, δ, γ) (40× magnification). Bar graphs representing relative protein expressions of PPARα, PPAR δ and PPAR γ to CPB, respectively (n = 6 per group, except the HFHF group, n = 5). The ratio of each PPAR subtype was expressed as mean ± SD. Scale bar was set as 200 µm. ^a^: *p* < 0.05 when compared with the control group, ^b^: *p* < 0.05 when compared with the ORX group, ^c^: *p* < 0.05 when compared with the ORX + HFHF group, and ^d^: *p* < 0.05 when compared with the HFHF group.

To confirm the alterations of PPAR subtypes expression in rat liver, the immunonohistochemistry analysis was performed in this study (Figure 7). Each of PPAR subtypes positive staining data was expressed as the ratio between the number of positive pixels to total number of pixels. The ORX + HFHF group showed significantly decreased in both PPARα and PPARδ positive staining when compared to the control group (PPARα: 0.47 ± 0.03 vs. 0.66 ± 0.05, PPARδ: 0.69 ± 0.09 vs. 0.91 ± 0.02, respectively; *p* < 0.05). While PPARδ positivity in the HFHF group was significantly reduced, PPARα positivity was unchanged when compared to normal controls (PPARδ: 0.77 ± 0.06 vs. 0.66 ± 0.05; *p* < 0.05). Administration of testosterone, estradiol and genistein could increase both PPARα and PPARδ positive staining compared with the ORX + HFHF group (PPARα: 0.71 ± 0.05 vs. 0.72 ± 0.05 vs. 0.66 ± 0.06 vs. 0.47 ± 0.03, respectively; *p* < 0.05). Conversely, a significant increase of PPARγ positive staining was observed in the ORX + HFHF group as compared with control and ORX + standard diet groups (0.14 ± 0.04 vs. 0.016 ± 0.01 vs. 0.029 ± 0.01, respectively; *p* < 0.05). As in the ORX + HFHF group, PPARγ expression in the HFHF group was significantly higher than in normal controls (0.06 ± 0.02 vs. 0.016 ± 0.01; *p* < 0.05). PPARγ expression in the testosterone-treated group remained significantly higher than that in the control group (0.07 ± 0.01 vs. 0.016 ± 0.01; *p* < 0.05). Both estradiol and genistein treatment significantly reduced PPARγ positivity compared with the ORX + HFHF group (0.036 ± 0.01 vs. 0.05 ± 0.01 vs. 0.14 ± 0.04, respectively; *p* < 0.05).

## 4. Discussion

### 4.1. Testosterone Deficiency and NASH Pathogenesis

In this study, we evaluated the effects of genistein and sex hormone supplementation on the modification of transcription factor PPARs (α, δ and γ) and their contribution to the NASH pathogenesis by using a testosterone-deficient rat model fed with an HFHF diet in an attempt to mimic the condition of NASH in transwomen. Besides its ability to modulate lipid metabolism and inflammation, testosterone has been found to serve many protective functions in human body; therefore, the lower levels of testosterone is associated with several metabolic syndrome [24].

Our results indicated that testosterone deficiency induced via bilateral orchidectomy caused a higher degree of hepatocyte injury and inflammation than in normal rats. Testosterone deficiency could be a contributing risk factor of NASH by promoting de novo lipogenesis and hepatic inflammation as evidenced by elevated SREBP1c and FAS gene expression, and reduced PPARα expression along with the augmentation of STAT1 inflammatory signaling pathway. Cai and his team found that the lack of testosterone hormone worsened lipid metabolism, increased inflammatory activity, oxidative stress, and apoptosis which was observed in castrated male pigs [7]. Prior in vivo study showed that FAS mRNA expression was significantly increased in mice fed with high-fat diet (HFD) plus ORX, while SREBP1c expression was not statistically different when compared to the HFD sham group [25]. It was proposed that the reduction of PPARα expression directly related with lipogenesis via the regulation of SREBP1c and FAS target genes.

Apart from its effects on lipogenic regulation, testosterone deficiency induced via bilateral orchidectomy could also promote low-grade inflammation by increasing serum interleukin-6 (IL-6) levels, and a trend toward increase in interleukin-1α,β (IL-1α and β) and TNF-α levels in ORX rats [26]. We hypothesized that testosterone deficiency might also trigger inflammation via the STAT1 pathway, leading to the development of NASH as shown by the increased trend of p-STAT1 expression in ORX rats fed standard diet in our study. Previous studies demonstrated that IFN-γ directly induced STAT1 activation and promoted hepatic inflammation in rats received HFD, while the deletion of STAT1 in liver macrophages resulted in loss of IFN- γ induction in physiological response [27,28]. Moreover, STAT1 and STAT3 activation were associated with early NAFLD development [18]. We hypothesized that testosterone deficiency is partly responsible for hepatic inflammation due to the activation of inflammatory chemokines.

### 4.2. High-Fat High-Fructose Diet Exacerbated NASH Development in Bilateral ORX Rats

This study was the first to elucidate that the presence of HFHF diet coupled with testosterone deficiency could induce a more severe form of NASH than HFHF diet alone. Our results showed that rats in the ORX + HFHF diet group had the highest degree of hepatic steatosis, lobular inflammation, hepatocyte ballooning, and percentage of positive Oil Red O staining area, including SREBP1c and FAS gene expression as compared to those of control, ORX + standard diet, and HFHF rats. Excessive consumption of fructose and saturated fat is associated with the buildup of liver fat, insulin resistance, and obesity, and they also contribute significantly to hepatic inflammation, thereby playing a key role in the development of NASH [29,30]. Fructose metabolism is recognized as a general pathway of hepatic de novo lipogenesis. The addition of fructose in the diet induces some transcriptional factors, i.e., carbohydrate responsive element binding protein (ChREBP) and SREBP1c, and also increases lipogenic gene expression when compared to HFD alone [31]. Additionally, fructose metabolism can account for ATP depletion, uric acid formation, oxidative stress production, and inflammation in the liver [29]. Since testosterone deficiency alone could increase de novo hepatic lipogenesis, it is not surprising that the combination of testosterone deficiency and HFHF diet consumption would lead to more severe hepatic steatosis and liver injury.

Dysregulation of transcription factors PPARs is assumed to play an important part in ORX + HFHF-induced NASH. The different isotypes of PPARs have been extensively studied in NAFLD and/or NASH conditions. Both PPARα and PPARδ seem to have similar functions. They primarily regulate the genes responsible for the breakdown of fatty acids through peroxisomal and mitochondrial β-oxidation and transcription factors that are involved in lipogenesis, inflammation, and fibrosis [32,33]. Francque et al. found that there was a correlation between reduced PPARα expression in the liver and elevated insulin resistance and a more severe NASH [34]. A previous study also showed that mice fed with a high-fat diet exhibited reduced levels of PPARδ mRNA in the liver [35]. In this study, HFHF diet alone led to the reduction in both PPARα and PPARδ expression. We also found lower PPARα and PPARδ expression in the ORX + HFHF diet group when compared to the HFHF group. In contrast, PPARγ exerts different effects from PPARα and PPARδ on NAFLD/NASH development. It was noteworthy that hepatic PPARγ and STAT1 expression were upregulated in NAFLD patients and in NAFLD/NASH animal models which were given the HFHF diet [14,36]. In addition, the upregulation of PPARγ led to the development of NAFLD development in mice fed with the HFHF diet through AMPK/Sirt1/SREBP-1c/PPARγ pathway [37]. Similar to other studies, the expression of PPARα and PPARδ were downregulated, whereas the expression of PPARγ and STAT1 were upregulated in the ORX + HFHF diet group in our study. We hypothesized that the interference of transcription factors PPARs (α, δ, γ) and STAT1 signal transduction activation were responsible for a more severe form of NASH found in testosterone deficient rats that also received the HFHF diet.

It should be noted that the delta body weight change in both HFHF alone and ORX + HFHF groups was not higher than the rats fed with standard diet groups. The results of other studies reported that the body weight of HFHF-diet-fed rats was decreased when compared to standard-diet-fed rats [38] and the delta body weight change in mice with NASH induced via a combination of high fructose in drinking water and HFD reduced when compared with the standard diet group [39]. Our hypothesis was that our diet regimen rich in fat and fructose composition could potentially result in a deficiency of essential amino acids, specifically methionine. This deficiency, in turn, could contribute to the development of hepatic steatosis, oxidative stress, and inflammation, despite the observed weight loss.

### 4.3. Sex Hormone Supplementation Attenuated Fat Accumulation and Inflammation, and Improved NASH Pathophysiology through the Modification of Hepatic PPARα,δ,γ and STAT1 in ORX + HFHF Rats

Testosterone and/or estradiol have beneficial effects in reducing body fat mass, lipid synthesis genes, oxidative stress and inflammation consequently leading to an improvement in NAFLD/NASH. Recent studies demonstrated that the absence of estrogen and testosterone hormone accelerated NAFLD development in rat models that underwent ovariectomy and orchidectomy together with being given an HFHF diet or high-cholesterol diet, respectively [11,20]. These manifestations were improved by male and female sex hormone therapies. In the current study, both testosterone and estradiol treated groups were set as positive controls. In order to simulate a scenario closely resembling humans, the standard dosage of testosterone enanthate and estradiol valerate was used to treat NASH [40,41]. We demonstrated that testosterone and estradiol improved overall NASH histological parameters, ameliorated hepatic fat accumulation, and decreased SREBP1c and FAS gene expression, compared with the ORX + HFHF group. Previous studies showed that the administration of exogenous estradiol and dihydrotestosterone (DHT) increased the expression of AR and ERα mRNA levels in ORX rats fed with HFD. This indicated that AR and ERα mediated the biological actions of sex hormones responsible their anti-lipogenesis effect through the reduction of fatty acid synthase (FAS) levels [11]. Alternatively, testosterone underwent aromatization in the body, resulting in the production of estradiol. Consequently, treatment with testosterone could produce an increase in serum estradiol levels and exerted its effects through estrogen receptors. Thus, our study confirmed that both testosterone and estradiol treatment had beneficial effects on hepatic lipid metabolism through direct SREBP1c and FAS gene regulation.

The potential mechanism through which anabolic steroids may impact the liver involves enhancing hepatic fatty acid oxidation by increasing hepatic PPARα, as well as promoting the activity of hepatic PPARδ, whereas PPARγ is responsible for controlling the energy homeostasis and regulating the storage of lipids. According to the study conducted by Zhang et al., PPARα gene expression was unchanged in both estradiol and DHT treated ORX rats fed with HFD [11]. On the other hand, another study suggested that anti-lipogenic effects of estradiol were partially attributed to the activation of PPARα and the inhibition of LXRα-dependent signaling pathways in rat livers [42]. A previous in vivo study demonstrated that the activation of NF-ĸB signaling pathway and the reduction of PPARδ expression played essential roles in NAFLD induced via testosterone deficiency and HFD in pigs, and hepatic steatosis and inflammation were alleviated after testosterone treatment [7]. Moreover, testicular feminized (Tfm) mice fed with a normal diet exhibited elevated gene expression of PPARγ, while FASN remained unchanged. However, when Tfm mice were supplemented with testosterone, there was a decrease in lipid accumulation in the liver compared to Tfm mice treated with a placebo. This reduction was attributed to a decrease in the expression of crucial regulatory enzymes involved in fatty acid synthesis [43]. These observations were in alignment with our findings that testosterone and estradiol treatment normalized NASH pathological features, restored PPARα and PPARδ expression, and reduced PPARγ protein levels comparing to the ORX + HFHF group. STAT1 has also been a target of sex hormone treatment. A previous in vitro study revealed that testosterone exerted an anti-inflammatory effect in lipopolysaccharide (LPS)-induced prostate cell inflammation by inhibiting pro-inflammatory cytokines (i.e., IL-1β, IL-6 and IL-8), and suppressing the IFNγ/STAT1 signaling pathway [44]. Moreover, immunoprecipitation analysis demonstrated that the recruitment of STAT1 to the promoter region of ERα is related to ERα transcription. This finding suggested that transcriptional regulation is one of the mechanisms through which STAT1 modulates the levels of ERα mRNA and ERα signaling in breast cancer cells [45]. Similar to other studies, our results showed that STAT1 expression in liver was decreased in testosterone and estradiol treated groups compared to the ORX + HFHF group, suggesting that sex hormone treatment might exert actions through the inhibition of STAT1 signaling pathway.

### 4.4. Genistein Ameliorated Fat Accumulation and Inflammation, and Improved NASH Pathophysiology through the Modification of Hepatic PPARα,δ,γ and STAT1 in ORX + HFHF Rats

Due to its structural-like estrogen molecule, genistein has become an alternative hormone replacement therapy for transgender women with NASH [46]. In this study, genistein had a lower effect on hepatic steatosis compared to estradiol because its affinity to estrogen receptors is weaker than estradiol [47]. Genistein shows potential as a dietary additive for regulating lipid accumulation in animals. According to other studies, genistein showed an anti-lipogenic effect by reducing hepatic FAS and SREBP1c expression, and increasing the levels of PPARα, estrogen receptor β (ERβ), SIRT1, and AMPK expression in both poultry and rodent models. These findings suggest lower rates of lipid synthesis and higher rates of β-oxidation in the liver in genistein-treated animals [48,49]. Apart from its estrogenic effects, genistein could induce the expression of PPARα, which subsequently regulated the pathways of fatty acid beta-oxidation in the liver. The activation of PPARα prevented the accumulation of triglycerides and has been associated with histological improvements in NASH in human studies [50]. Similar to our findings, Zengpeng and colleagues demonstrated that dietary genistein supplementation resulted in the upregulation of gene transcription associated with fatty acid beta-oxidation, including PPARα, PPARδ, ACOT8, ACAD8, and ACADs in the liver of laying hens induced fatty liver. Additionally, this treatment led to a reduction in the expression of PPARγ and AFABP in abdominal fat [15]. STAT1 is another key mediator of chronic inflammatory response and is activated in chronic liver diseases [51]. An in vitro study by Jantaratnotai et al. found that treatment with genistein (1 μM) significantly inhibited the upregulation of iNOS, IRF-1, and p-STAT1 protein expression induced via LPS-activated microglia [52]. Moreover, dietary genistein had the potential to directly alleviate the inflammatory response by modulating the expression of inflammatory factors such as NF-кB, IL-8, IL-1β, and IFN-γ through the involvement of PPARδ and T-cell factor 3 (TCF3) in laying hens induced fatty liver [15]. Therefore, the inhibition of STAT1 activation by IFN-γ receptor may be responsible for the immune-modulatory effects of genistein in NASH; however, the precise interactions between STAT1 and IFN-γ required further investigation.

For this study, the dose of genistein at 16 mg/kg BW was based on our previous publications, which demonstrated its efficacy in improving NASH induced via ovariectomy (OVX) plus HFHF diet in female rats. This dosage of genistein may not have the same protective effects against NASH as sex hormones do. Using this study as a starting point for applying genistein in the treatment of NASH in transgender patients, further exploration of higher dosages of genistein would be a promising avenue for future research.

## 5. Conclusions

**Figure 8:**
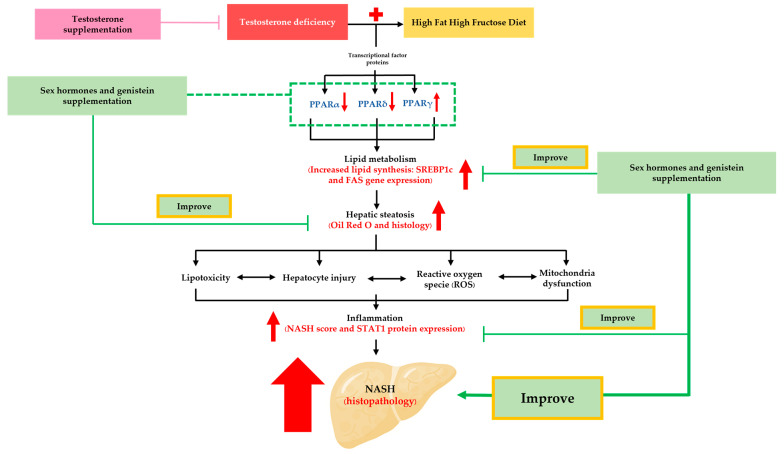
An overview diagram of the effects of genistein and sex hormone supplementation on a NASH rat model with bilateral orchidectomy induced via an HFHF diet. The pathology of NASH worsened after a six-week consumption of a high-fat high-fructose diet and testosterone deficiency. However, testosterone, estradiol, and genistein showed their beneficial effects in ameliorating NASH pathogenesis through anti-lipogenic and anti-inflammatory properties. This was evidenced by the attenuation in FAS and SREBP1c mRNA expression, the regulation of these PPARα, δ, and γ subtypes expression, and the inhibition of STAT1 expression.

In summary, we provided evidence in Figure 8 that testosterone deficiency induced via orchidectomy aggravated the severity of NASH in the setting of a HFHF diet. Testosterone, estradiol, and genistein supplementation could hinder the development of NASH through the reduction of SREBP1c and FAS gene transcription, the activation of PPARα and PPARδ expressions, and the suppression of PPARγ and STAT1. These findings provided more insight into possible molecular mechanism underlying the association between transcription factors (PPARs) and STAT1 pathway and suggested that genistein might be useful as a hormone replacement therapy to prevent NASH in transgender women. Moreover, clinical studies are required to determine the suitable dosage and safety of genistein in transgender women with NASH.

## Figures and Tables

**Table 1:** The overview of liver histological scores of steatosis, lobular inflammation, and hepatocellular ballooning following Brunt’s criteria.

Group	n	Steatosis				Lobular Inflammation				Hepatocyte Ballooning			NASH Activity Score
		0	1	2	3	0	1	2	3	0	1	2	
Control	6	6	-	-	-	4	2	-	-	3	3	-	0.83 ± 0.30
ORX	6	6	-	-	-	-	5	1	-	2	3	1	2.00 ± 0.44
ORX + HFHF	6	-	1	2	3	-	2	4	-	-	1	5	5.50 ± 0.42 ^a,b^
HFHF	5	1	2	2	-	-	3	2	-	-	2	3	4.20 ± 0.20 ^a,b^
ORX + HFHF + Testosterone	6	4	2	-	-	5	1	-	-	1	3	2	1.83 ± 0.30 ^c,d^
ORX + HFHF + Estradiol	6	5	1	-	-	6	-	-	-	-	1	5	2.00 ± 0.00 ^c,d^
ORX + HFHF + Genistein	6	1	3	1	1	2	4	-	-	-	3	3	3.50 ± 0.34 ^a,b,c,e,f^

Data are shown as the number of rats demonstrating each score of liver histological grading. The summation of NASH activity score is reported as mean ± SD. ^a^: *p* < 0.05 when compared with the control group, ^b^: *p* < 0.05 when compared with the ORX group, ^c^: *p* < 0.05 when compared with the ORX + HFHF group, ^d^: *p* < 0.05 when compared with the HFHF group, ^e^: *p* < 0.05 when compared with the ORX + HFHF + Test group and ^f^: *p* < 0.05 when compared with the ORX + HFHF + E2 group. Note: During week 6, one rat from the HFHF group died due to an unknown cause, resulting in a total of five rats remaining in the HFHF group.

## Data Availability

The data that support the findings of this study are available from the corresponding author, [D.W.], upon reasonable request.

## Supplementary Materials

The following supporting information can be downloaded at: https://www.mdpi.com/article/10.3390/biomedicines12030483/s1. Supplementary Table S1. Specific primers sequence for gene expression analysis. Supplementary Table S2. The quantification data of Oil Red O staining area presented as a percentage in all experimental groups. Supplementary Figure S1. The bilateral orchidectomy procedure was performed to induce the state of testosterone deficiency in orchidectomized groups. Supplementary Figure S2. Weekly mean body weight growth curves of the rats in all experimental groups (n = 6 per group, except HFHF group, n = 5). Supplementary Figure S3. Immunoblotting images for PPARα and cyclophilin B (CPB) band densities. Supplementary Figure S4. Immunoblotting images for PPARδ and cyclophilin B (CPB) band densities. Supplementary Figure S5. Immunoblotting images for PPARγ and cyclophilin B (CPB) band densities. Supplementary Figure S6. Immunoblotting images for p-STAT1 and t-STAT1, and cyclophilin B (CPB) band densities.
