# Supplementary material for: Genistein and Sex Hormone Supplementation Modulated Hepatic PPARα, δ, and γ Subtypes and STAT1 Expressions in a NASH Rat Model with Bilateral Orchidectomy
Source: Biomedicines. 2024 Feb 21;12(3):483. doi: 10.3390/biomedicines12030483 (PMC10968543; doi:10.3390/biomedicines12030483)
Supplement: Supplementary file 1 [file biomedicines-12-00483-s001.zip › biomedicines-2818559-supplementary.pdf]

Title: Genistein and Sex Hormone Supplementation Modulated Hepatic PPARα, δ and γ Subtypes and STAT1 Expressions in a NASH Rat Model with Bilateral Orchidectomy

Authors: Fatist Okrit, Maneerat Chayanupatkul, Prasong Siriviriyakul, Natcha Wanpiyarat and Duangporn Werawatganon*

Type of manuscript: Original Research Article

Supplementary Table S1. Specific primers sequence for gene expression analysis.

Gene	Function	Nucleotide sequence
FAS	Forward<br>reverse	5'-ACC-TCA-TCA-CTA-GAA-GCC-ACC-AG-3'<br>5'-GTG-GTA-CTT-GGC-CTT-GGG-TTT-A-3'
SREBP1c	Forward<br>reverse	5'-CTG-TCG-TCT-ACC-ATA-AGC-TGC-AC-3'<br>5'-ATA-GCA-TCT-CCT-GCA-CAC-TCA-GC-3'
β-actin	Forward<br>reverse	5'-ACG-GTC-AGG-TCA-TCA-CTA-TCG-3'<br>5'-GGC-ATA-GAG-GTC-TTT-ACG-GAT-G-3'

Supplementary Table S2. The quantification data of Oil Red O staining area presented as a percentage in all experimental groups.

Groups / Lipid deposition	Control	ORX	ORX+HFHF	HFHF	ORX+HFHF+ Test	ORX+HFHF+ E2	ORX+HFHF+ Gen
Percentage of positive Oil Red O staining area	2.80±0.98	6.68±1.30[a]	54.19±1.88[a,b]	26.9±4.20[a,b,c]	2.86±0.36[b,c,d]	2.03±0.70[b,c,d]	10.77±2.99[a,b,c,d]

Data is expressed as mean ± SD. a: $p<0.05$ when compared with the control group, b: $p<0.05$ when compared with the ORX group, c: $p<0.05$ when compared with the ORX+HFHF group, d: $p<0.05$ when compared with the HFHF group.

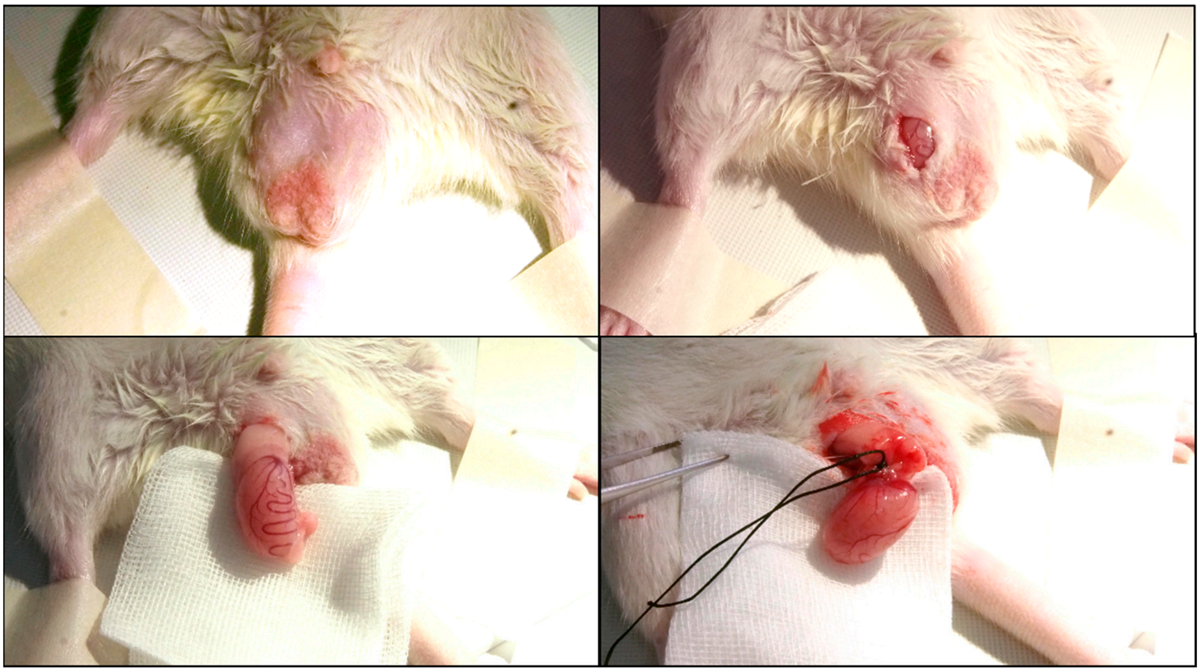

Supplementary Fig S1. The bilateral orchidectomy procedure was performed to induce the state of testosterone deficiency in orchidectomized groups.

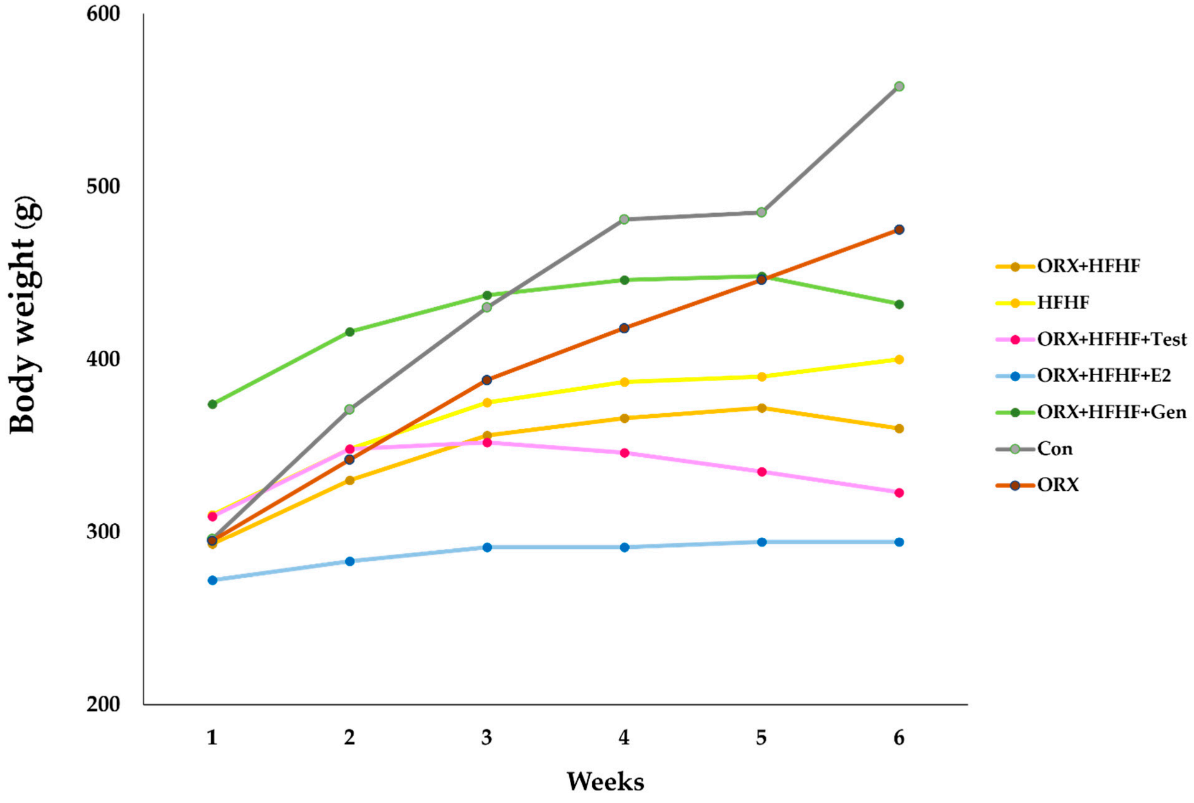

Supplementary Fig S2. Weekly mean body weight growth curves of the rats in all experimental groups (n=6 per group, except HFHF group, n=5).

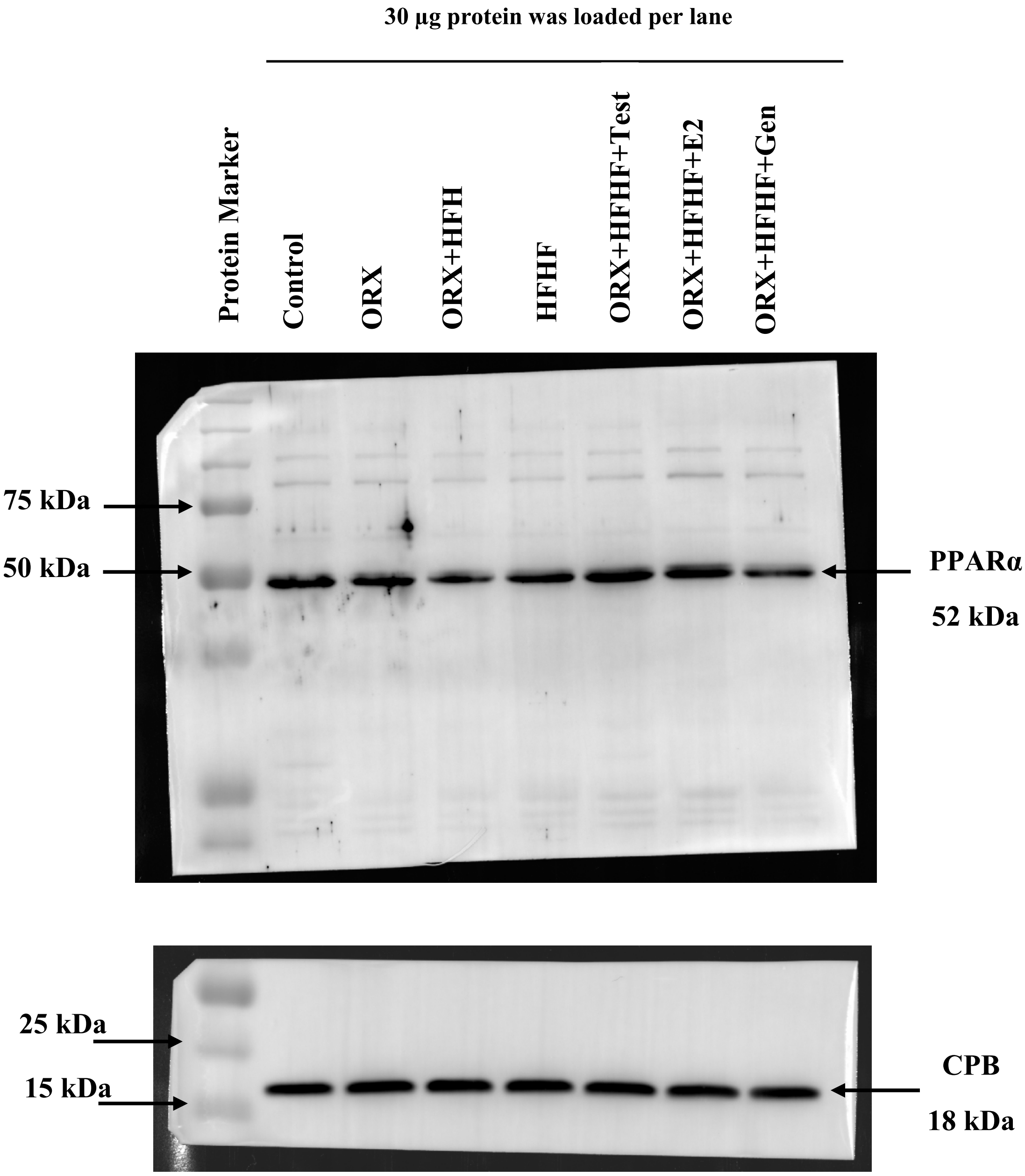

Supplementary Fig S3. Immunoblotting images for PPAR*α* and cyclophilin B (CPB) band densities. Seven panels (Left to right side) indicate each experimental group. The first four panels represent untreated groups; control, ORX, ORX+HFHF and HFHF groups, respectively and the last three panels represent all treated groups; ORX+HFHF+Test, ORX+HFHF+E2 and ORX+HFHF+Gen groups, respectively). Abbreviations: Test, Testosterone; E2, estradiol; Gen, Genistein.

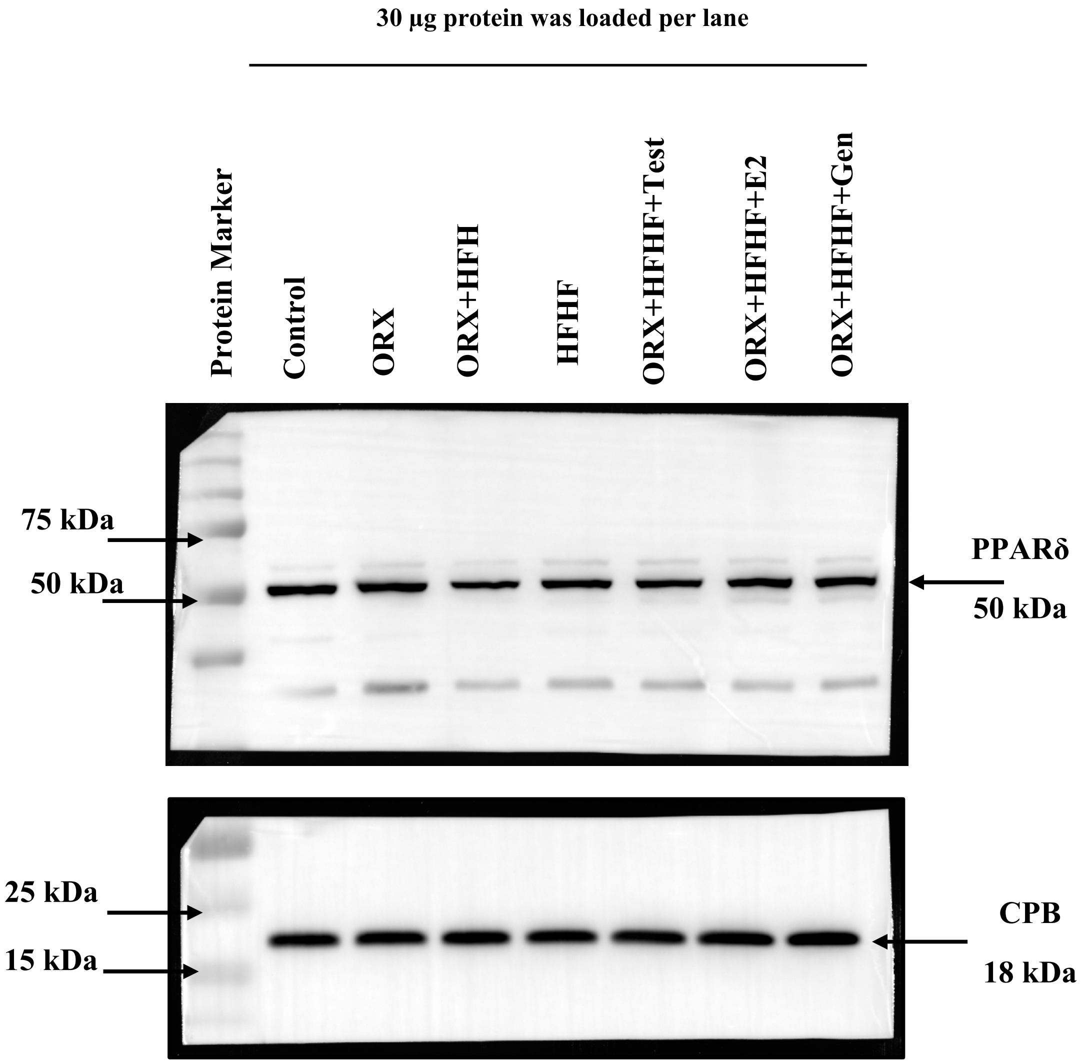

Supplementary Fig S4. Immunoblotting images for PPARδ and cyclophilin B (CPB) band densities. Seven panels (Left to right side) indicate each experimental group. The first four panels represent untreated groups; control, ORX, ORX+HFHF and HFHF groups, respectively and the last three panels represent all treated groups; ORX+HFHF+Test, ORX+HFHF+E2 and ORX+HFHF+Gen groups, respectively). Abbreviations: Test, Testosterone; E2, estradiol; Gen, Genistein.

30 µg protein was loaded per lane

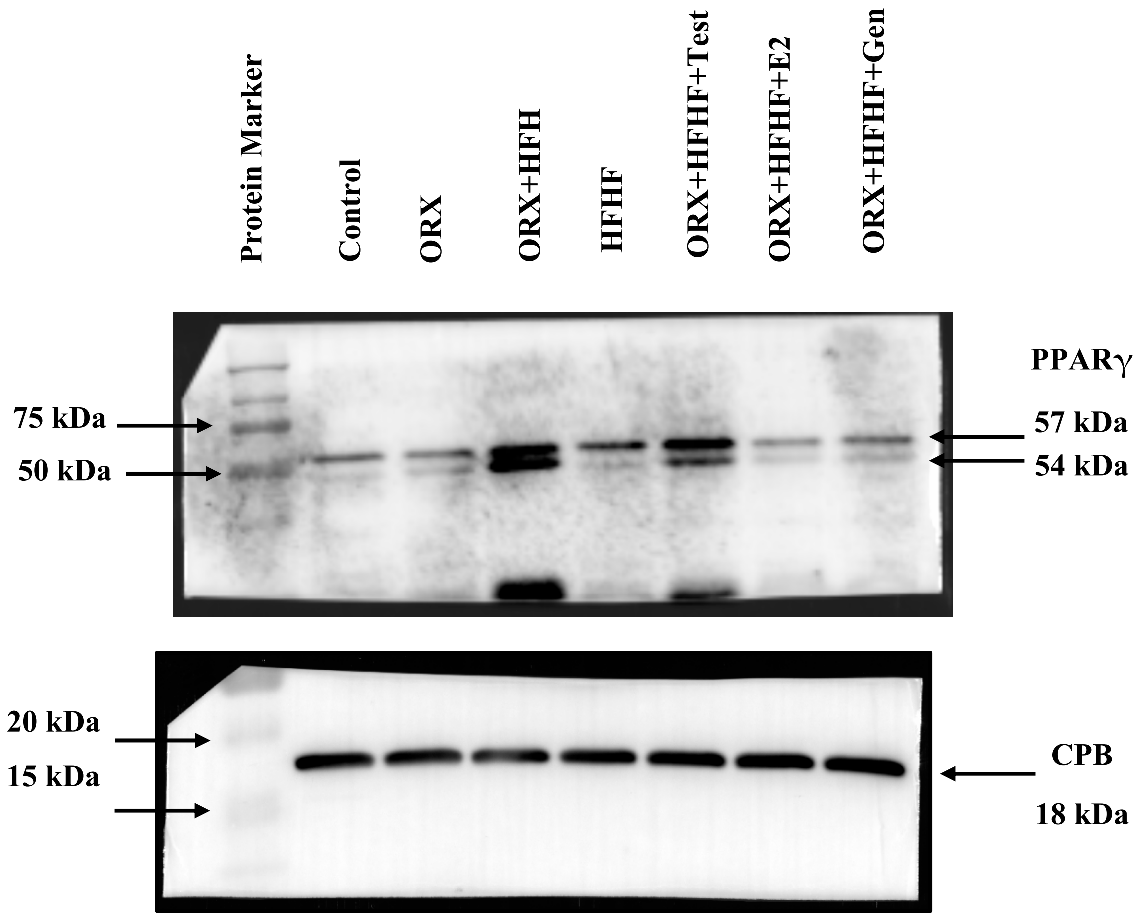

Supplementary Fig S5. Immunoblotting images for PPARγ and cyclophilin B (CPB) band densities. Seven panels (Left to right side) indicate each experimental group. The first four panels represent untreated groups; control, ORX, ORX+HFHF and HFHF groups, respectively and the last three panels represent all treated groups; ORX+HFHF+Test, ORX+HFHF+E2 and ORX+HFHF+Gen groups, respectively). Abbreviations: Test, Testosterone; E2, estradiol; Gen, Genistein.

30 µg protein was loaded per lane

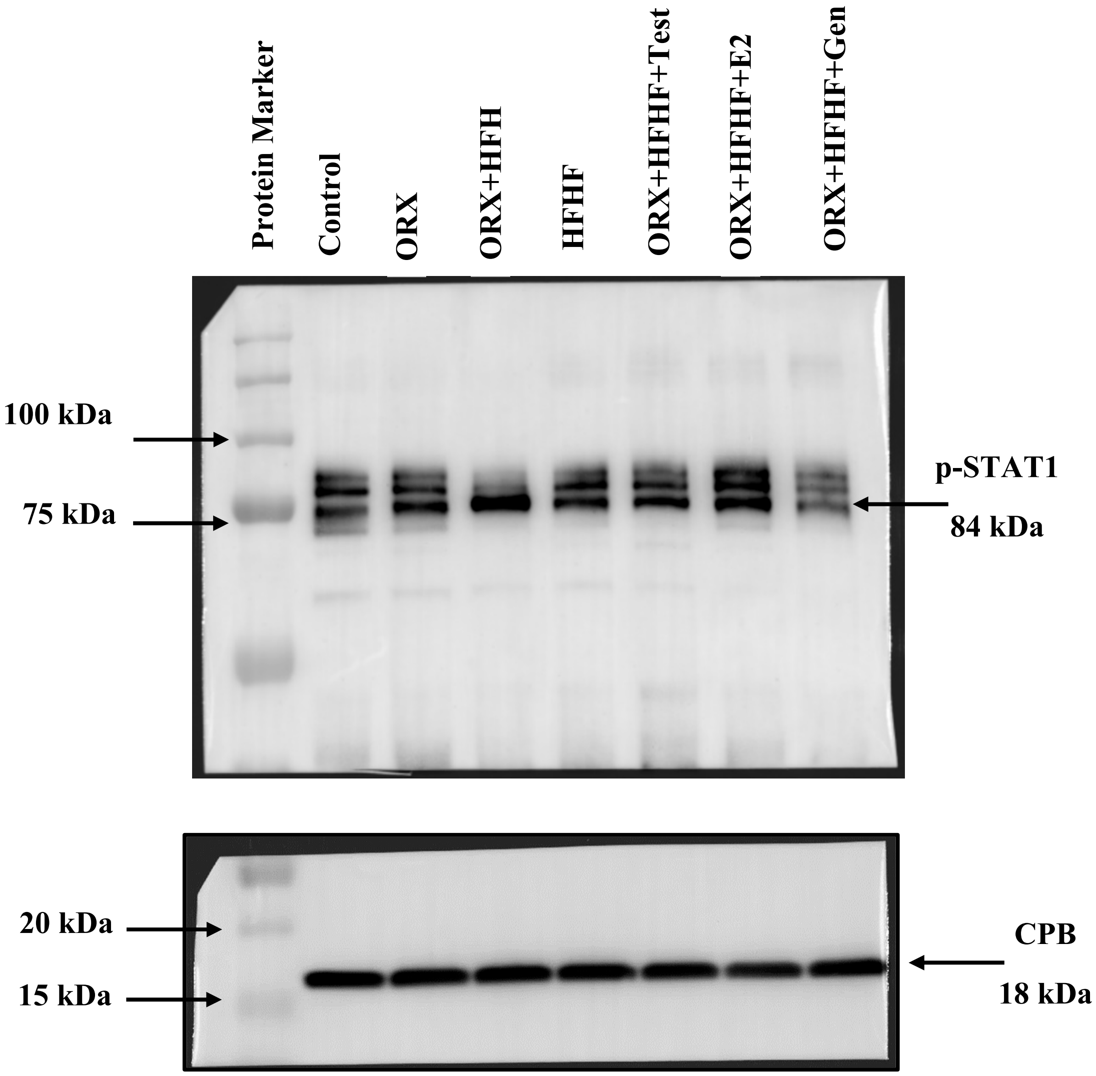

30 µg protein was loaded per lane

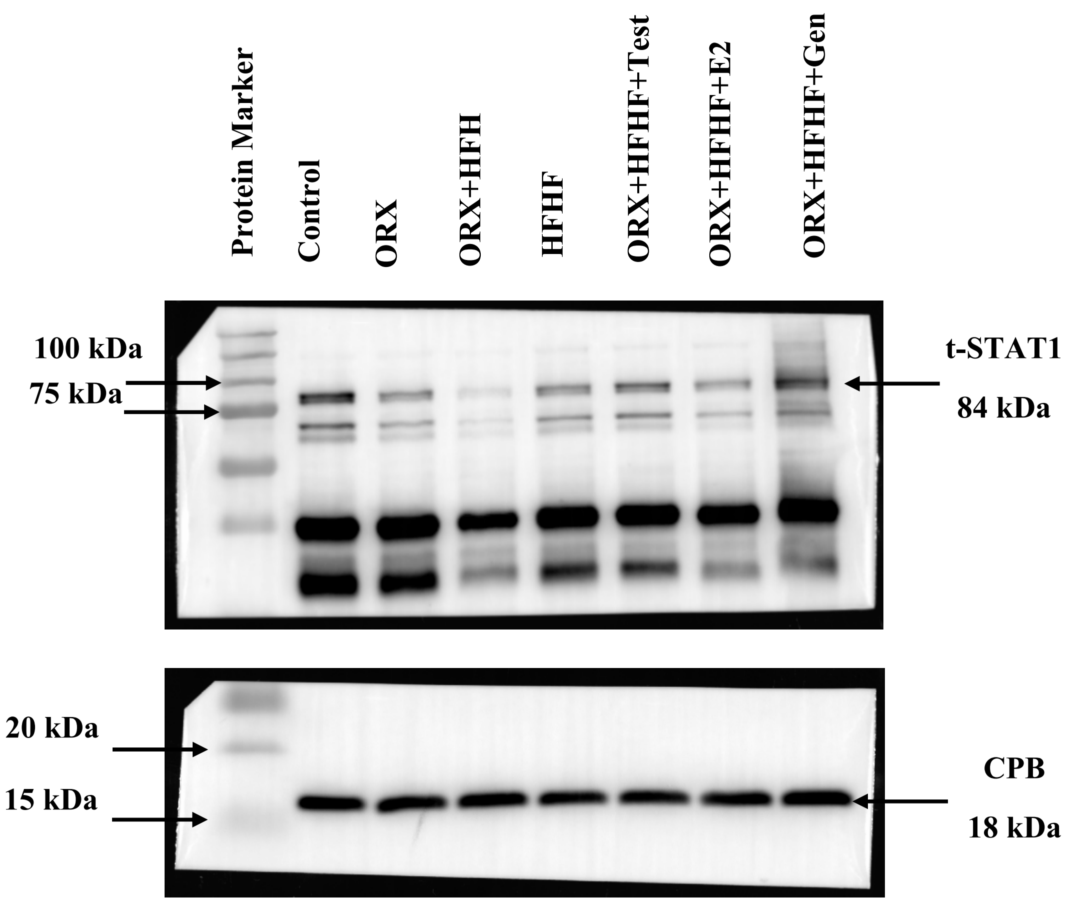

Supplementary Fig S6. Immunoblotting images for p-STAT1 and t-STAT1, and cyclophilin B (CPB) band densities. Seven panels (Left to right side) indicate each experimental group. The first four panels represent untreated groups; control, ORX, ORX+HFHF and HFHF groups, respectively and the last three panels represent all treated groups; ORX+HFHF+Test, ORX+HFHF+E2 and ORX+HFHF+Gen groups, respectively). Abbreviations: Test, Testosterone; E2, estradiol; Gen, Genistein.
